# ICU bereaved surrogates’ comorbid psychological-distress states and their associations with prolonged grief disorder

**DOI:** 10.1186/s13054-022-03981-7

**Published:** 2022-04-11

**Authors:** Fur-Hsing Wen, Wen-Chi Chou, Chung-Chi Huang, Tsung-Hui Hu, Ming Chu Chiang, Li-Pang Chuang, Siew Tzuh Tang

**Affiliations:** 1 Department of International Business, Soochow University, Taipei, Taiwan, ROC; 2 Division of Hematology-Oncology, Chang Gung Memorial Hospital at Linkou, Tao-Yuan, Taiwan, ROC; 3 College of Medicine, Chang Gung University, Tao-Yuan, Taiwan, ROC; 4 Department of Internal Medicine, Division of Pulmonary and Critical Care Medicine, Chang Gung Memorial Hospital at Linkou, Tao-Yuan, Taiwan, ROC; 5 Department of Respiratory Therapy, Chang Gung University, Tao-Yuan, Taiwan, ROC; 6 Department of Internal Medicine, Division of Hepato-Gastroenterology, Chang Gung Memorial Hospital at Kaohsiung, Kaohsiung, Taiwan, ROC; 7 Department of Nursing, Chang Gung Memorial Hospital at Kaohsiung, Kaohsiung City, Taiwan, ROC; 8 School of Nursing, Medical College, Chang Gung University, 259 Wen-Hwa 1st Road, Kwei-Shan, Tao-Yuan, 333 Taiwan, ROC

**Keywords:** Psychological distress, Bereavement, Anxiety, Depression, Post-traumatic stress disorder, Prolonged grief disorder, End-of-life care, ICU care

## Abstract

**Background/objective:**

Bereaved ICU family surrogates’ psychological distress, e.g., anxiety, depression, and post-traumatic stress disorder (PTSD), is usually examined independently, despite the well-recognized comorbidity of these symptoms. Furthermore, the few studies exploring impact of psychological distress on development of prolonged grief disorder (PGD) did not consider the dynamic impact of symptom evolution. We identified surrogates’ distinct patterns/states of comorbid psychological distress and their evolution over the first 3 months of bereavement and evaluated their associations with PGD at 6-month postloss.

**Methods:**

A longitudinal observational study was conducted on 319 bereaved surrogates. Symptoms of anxiety, depression, PTSD, and PGD were measured by the anxiety and depression subscales of the Hospital Anxiety and Depression Scale, Impact of Event Scale-Revised scale, and the PGD-13, respectively. Distinct psychological-distress states and their evolution were examined by latent transition analysis. Association between psychological-distress states and PGD symptoms was examined by logistic regression.

**Results:**

Three distinct comorbid psychological-distress states (prevalence) were initially identified: no distress (56.3%), severe-depressive/borderline-anxiety distress (30.5%), and severe-anxiety/depressive/PTSD distress (13.3%). Except for those in the stable no-distress state, surrogates tended to regress to states of less psychological distress at the subsequent assessment. The proportion of participants in each psychological-distress state changed to no distress (76.8%), severe-depressive/borderline-anxiety distress (18.6%), and severe-anxiety/depressive/PTSD distress (4.6%) at 3-month postloss. Surrogates in the severe-depressive/borderline-anxiety distress and severe-anxiety/depressive/PTSD-distress state at 3-month postloss were more likely to develop PGD at 6-month postloss (OR [95%] = 14.58 [1.48, 143.54] and 104.50 [10.45, 1044.66], respectively).

**Conclusions:**

A minority of family surrogates of ICU decedents suffered comorbid severe-depressive/borderline-anxiety distress and severe-anxiety/depressive/PTSD symptoms during early bereavement, but they were more likely to progress into PGD at 6-month postloss.

**Supplementary Information:**

The online version contains supplementary material available at 10.1186/s13054-022-03981-7.

## Background

Psychological distress among family members of critically ill patients is well recognized [1, 2]. Still, few studies examined bereaved family members [1] despite their experiences of the uncertain trajectory of critical illness, the highly emotion-laden end-of-life (EOL)-care decision-making process, frightening aggressive life-sustaining treatments, and the patient’s eventual death. With these factors combined, family members of intensive care unit (ICU) decedents face a stressful and traumatic experience [3]. Thus, bereaved family members commonly suffer clinically significant psychological distress [1, 2], including anxiety [4–15], depression [4–19], post-traumatic stress disorder (PTSD) [4, 6–20], and prolonged/complicated grief disorder (PGD) [5, 6, 9, 12, 13, 18–21]. Such psychological distress takes a toll on personal psychological and physical well-being [22–26] and social functioning [25, 27], and imposes a financial burden for individuals, healthcare systems, and society [24, 26, 28, 29]. Thus, to improve EOL-care quality in ICUs, an international priority for critical care [30], understanding the psychological burden of critical illness and ICU caregiving on family members of ICU decedents is essential [31].

Among the studies on bereaved family members of ICU decedents, symptoms of anxiety, depression, and PTSD were usually examined independently, except for two studies focused on co-occurrence of anxiety or depression with PTSD [4, 16], despite the well-recognized comorbidity of depression and anxiety [32]; depression and PTSD [33]; and anxiety, depression, and PTSD [32]. Therefore, the collective psychological burden of ICU decedents’ family members may be better understood by exploring comorbid anxiety/depression/PTSD symptoms and identifying them as patterns of psychological distress (“latent states”).

Furthermore, while most bereaved people recover with time, a minority maladaptively adjust to their loss [34], their distress evolving into PGD—unrelenting emotional distress beyond normal grief [25]. However, few studies explored the co-occurrence or impact of symptoms of anxiety, depression, or PTSD on the development of PGD. PTSD and complicated/prolonged grief were shown to co-occur among bereaved family members of ICU decedents at 6-month postloss [6, 9, 19]. Symptoms of anxiety, depression, or PTSD were examined univariately as associated [9, 19] or not associated with PGD [6] concurrently [6] or prospectively [6, 9, 19]. When the prospective associations between PGD and symptoms of anxiety, depression or PTSD were examined, predisposing symptoms were measured at 1 [6] or 3 [9, 19] months postloss only, overlooking the fluid nature of those symptoms [6, 9, 12, 13]. Therefore, the purposes of this study were to identify ICU bereaved family surrogates’ distinct patterns (states) of psychological distress (i.e., comorbid symptoms of anxiety, depression, and PTSD) and their evolution over the first 3 months of bereavement and to evaluate their associations with the development of PGD symptoms at 6-month postloss. Bereaved surrogates’ symptoms of anxiety [35], depression [35], and PTSD [36] have been individually reported. Herein, we extend our understanding of bereaved surrogates’ individual psychological distress into their comorbid states.

## Methods

### Study design/setting/study participants

This study is part of a longitudinal, observational study on associations between quality of EOL care in ICUs and family surrogates’ bereavement outcomes [35, 36]. Sampling strategy and characteristics of the study settings were reported [35, 36]. Briefly, ICU patients who were identified at enrollment as high risk for dying by Acute Physiology and Chronic Health Evaluation (APACHE) II score ≥ 20 were recruited consecutively from level III medical ICUs staffed by intensivists in two academically affiliated hospitals in Taiwan. Patients who died within 3 days of ICU admission were excluded to allow sufficient time to implement high-quality EOL care [9]. Family members who self-identified as legally authorized to be the patient’s surrogate for his/her medical decisions and who were cognitively competent to be interviewed were recruited consecutively from January 2018 to January 2020 and followed through June 2021. Only one family surrogate who took primary EOL-care decision-making responsibility was recruited per patient. Each surrogate signed informed consent for their participation and for allowing review of the patient’s medical record. The research ethics committee of the study site approved the research protocol (201700343B0).

### Data collection

Patients’ and family surrogates’ demographics were recorded at enrollment. Experienced, trained research assistants phone interviewed family surrogates to assess their symptoms of anxiety, depression, and PTSD at 1- and 3-month postloss, and PGD symptoms at 6-month postloss to comply with the duration criterion for PTSD [37] and PGD [25]. Phone calls were made during different periods over a week (e.g., morning and evening, different weekdays) if the first attempt failed to reach participants. The time-window for assessments of psychological distress was set for 2 weeks to maintain consistency with pre-set bereavement survey time points. The principal investigator provided pre-service trainings to ensure inter-rater reliability of data collections by phone-interviews, conducted regular team meetings to discuss any issues/concerns with data collections, and periodically checked the consistency of data collected.

### Measures

*Anxiety and depressive symptoms* were measured by the Hospital Anxiety and Depression Scale (HADS) [38]. Seven HADS items measure anxiety (HADS-A subscale) and depression (HADS-D subscale), respectively, and each has a total score ranging from 0 to 21. Severe anxiety and depressive symptoms were identified as HADS-A and HADS-D subscale scores ≥ 8.

*PTSD symptoms* were measured by the 22-item Impact of Event Scale-Revised (IES-R) [37]. Each item is rated for PTSD-related symptom distress level during the preceding week on a 0 (not at all)-4 (extremely) Likert scale. IES-R scores ≥ 33 indicate severe PTSD symptoms [37].

*PGD symptoms* were assessed with the PGD-13 [25]. To be categorized as PGD, the following criteria must be met: the experience of yearning, and the daily or disabling experience of at least five of nine symptoms (feeling emotionally numb, stunned, that life is meaningless; perceiving the future as purposeless or meaningless; experiencing mistrust; bitterness over the loss; difficulty accepting the loss; identity confusion; avoiding the reality of the loss; or difficulty moving on with life). Furthermore, symptoms must be present at sufficiently high levels ≥ 6-month postloss and be associated with functional impairment.

### Statistical analysis

A latent transition analysis (LTA) with hidden Markov modeling [39] was conducted to simultaneously identify family surrogates’ distinct states of psychological distress indicated by comorbid severe symptoms of anxiety, depression, and PTSD and to determine probabilities of shifting from one state to another between consecutive times (transition probability) over the first 3-month postloss using Latent GOLD 5.0. By using LTA, surrogates were assigned to a finite number of mutually exclusive probabilistic comorbid psychological-distress states based on characteristics shared by surrogates in each state, thus discriminating them from surrogates in other states. Emission probability signified the observed probability that each surrogate had or did not have severe symptoms of anxiety, depression, and PTSD in each identified state, conditional on his/her state membership [39]. Multiclass model solutions with an increasing number of states were assessed for their goodness of fit to the observed data by information criterion (IC). ICs are measurements used to compare multiple multiclass models for their ability to explain an observed dataset [40]. Smaller IC values indicate a better model fit [40]. The simplest model with the most explanatory power is best. As suggested by researchers [40, 41], best model solutions were selected by the following criteria: (1) model-fit indices of highest log-likelihood (LL) as well as smallest Akaike information criterion (AIC), Bayesian information criterion (BIC), and sample-size adjusted BIC (SABIC) [40] with more weight on flattening IC values between consecutive numbers of states in plots of IC value *vs*. state number [41], (2) highest entropy (a measure of certainty in class membership assignment, ranging between 0 and 1), (3) lowest classification error (a measure of errors in classification, ranging between 0 and 1); (4) parsimony, and (5) substantive clinical meaningfulness of the latent class identification.

The second part of LTA estimated state-transition probabilities [39]. Transition probability represents the likelihood that a surrogate had a comorbid psychological-distress state at time t, given his/her specific state at time t − 1 [39]. We estimated the probability for each comorbid psychological-distress state at 3-month postloss based on each state’s initial probability (size/prevalence) and transition probabilities. We used these lagged comorbid psychological-distress states to arrange a distinct time sequence when we examined associations between this clinically modifiable variable and each participant’s PGD at 6-month postloss by logistic regression modeling. Examining associations of the lagged comorbid psychological-distress states measured at 3-month postloss with subsequent development of PGD at 6-month postloss not only establishes the temporal relationship between them but also investigates the immediate (most proximal) effect of comorbid psychological-distress states on the development of PGD rather than the more distal effect from 1 month postloss. The regression estimate in the logistic regression models was exponentiated to transform into odds ratio (OR) with 95% confidence interval (CI).

## Results

### Participant characteristics

**Fig. 1:**
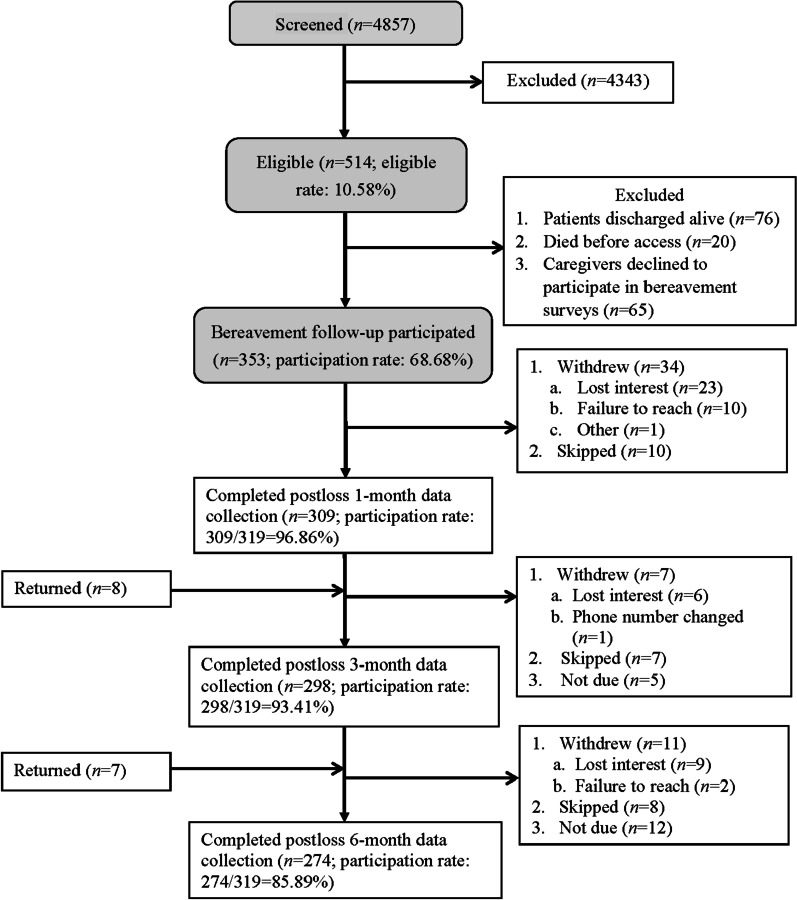
Participant flow chart

Among the 353 patients who died in the ICUs, 319 family surrogates (90.4%) participated in bereavement surveys and constituted the study participants (Fig. 1). Characteristics of these study participants and their loved ones are in Table 1. Among study participants, 309, 298, and 274 completed surveys at 1-, 3-, and 6-month postloss, respectively. No significant differences in patient or family demographics for participants and non-participants of bereavement surveys were reported [36] nor were significant differences observed among those who completed, skipped, or withdrew from postloss follow-ups over the first 6-month of bereavement (Additional file 1: Online Data Supplement 1).

**Table 1:** Characteristics of participants (*N* = 319)

Variable	*n*	%
**Family surrogates**		
Gender		
Male	130	40.8
Female	189	59.2
Relationship with the patient		
Spouse	94	29.5
Adult child	173	54.2
Others	52	16.3
Marital status		
Non-married	78	24.5
Married	241	75.5
Educational level		
> High school	158	49.5
≤ High school	161	50.5
Financial sufficiency		
Yes	268	85.9
No	44	14.1
Preexisting mental health and medical problems in the past year		
Hospitalization due to medical problems		
Yes	14	4.4
No	305	95.6
Hospitalization due to mental health problems		
Yes	0	0.0
No	319	100.0
Emergency room visits		
Yes	22	6.9
No	297	93.1
Use of medications for problems with pain		
Yes	35	11.0
No	284	89.0
Use of medications for problems with anxiety		
Yes	8	2.5
No	311	97.5
Use of medications for problems with depression or other psychiatric disturbances		
Yes	3	0.9
No	316	99.1
Age (mean [SD])	49.86 [12.52]	
**Patients**		
Gender		
Male	203	63.6
Female	116	36.4
Primary disease		
Cancer	160	50.2
Pulmonary	22	6.9
Cardiovascular	15	4.7
Kidney	16	5.0
Other	106	33.2
Acute symptoms/problems at admission		
Respiratory failure/distress	166	52.0
Infection	90	28.2
Shock	24	7.5
Cardiac arrest	12	3.8
Others	27	8.5
Comorbidity		
Yes	271	85.0
No	48	15.0

Variable	Median	Interquartile range
Age (years, mean [SD])	66.67 (14.22)	
APACHE^a^	28.0	24–32
Length of ICU stay (days)	17.0	10–29
Time from enrollment to death (days)	4.0	2–9

*SD* Standard deviation; *APACHE* Acute Physiology and Chronic Health Evaluation; *ICU* Intensive Care Unit

^a^Measure of disease severity at enrollment

### States of comorbid psychological distress identified by latent transition analysis and their evolution over the first 3 months of bereavement

The model-fit indices for the one through four-state LTA solutions of psychological distress indicated by comorbid severe symptoms of anxiety, depression, and PTSD are in Additional file 1: Online Data Supplement 2. A three-state solution was evaluated as most optimal and parsimonious by the AIC, BIC, SABIC, plots of IC values *vs*. state number (Additional file 1: Online Data Supplement 3), entropy, classification error, and clinical meaningfulness. Sizes (state prevalence) and emission probabilities of these three states are in Table 2. Visible differences in emission probabilities determined comorbidity of each state, and three identified states were named (prevalence): no distress (56.3%), severe-depressive/borderline-anxiety distress (30.5%), and severe-anxiety/depressive/PTSD distress (13.3%). Nil (0–2.9%) and nearly all (84.4–97.4%) family surrogates in the no-distress and the severe-anxiety/depressive/PTSD-distress state experienced severe symptoms of anxiety, depression, and PTSD, respectively, as shown by the emission probabilities. Surrogates in the severe-depressive/borderline-anxiety-distress state experienced severe symptoms of anxiety (38.3%), depression (88.2%), and PTSD (0.1%) (Table 2). Correspondingly, none and all of the distress symptoms among surrogates in the no-distress and the severe-anxiety/depressive/PTSD-distress states, respectively, exceeded the clinically significant thresholds (Additional file 1: Online Data Supplement 4). Mean scores of the HADS-D and HADS-A subscale for surrogates in the severe-depressive/borderline-anxiety-distress state exceeded and approached the thresholds, respectively; whereas scores for the IES-R were well below the threshold at 1- and 3-month postloss.

**Table 2:** Sizes and emission probabilities of the three-state solution of comorbid psychological distress for bereaved surrogates

Psychological distress (%)	Comorbid psychological-distress state	No distress	Severe-depressive/borderline-anxiety distress	Severe anxiety/depressive/PTSD distress
	1-month-postloss size (%)	56.3	30.5	13.3
	3-month-postloss size (%)	76.8	18.6	4.6
Severe anxiety symptoms				
Yes		0.0	38.3	85.0
No		100.0	61.7	15.0
Severe depressive symptoms				
Yes		2.9	88.2	97.4
No		97.1	11.8	2.6
Severe PTSD symptoms				
Yes		0.0	0.1	84.4
No		100.0	99.9	15.6

*PTSD* post-traumatic stress disorder

Transition probabilities estimated from the LTA showed that almost all surrogates (98.7%) in the no-distress state remained in their original state at the subsequent assessment (Table 3), indicating highly stable psychological status over their first 3 months of bereavement. In contrast, surrogates in the severe-depressive/borderline-anxiety-distress and the severe-anxiety/depressive/PTSD-distress states primarily shifted to no-distress and severe-depressive/borderline-anxiety-distress states, respectively; whereas 33.8–43.3% of surrogates in these two states remained in their original state at 3-month postloss. Ranking of comorbid psychological-distress states was the same at 3-month-postloss assessment based on LTA estimations, but the proportion of participants in each psychological-distress state changed: no distress (76.8%), severe-depressive/borderline-anxiety distress (18.6%), and severe-anxiety/depressive/PTSD distress (4.6%) (Table 2).

**Table 3:** Transition probabilities of different emotional-distress states from time [*t* − 1] to time [t]

Time [*t* − 1]		Time [t]	
Comorbid psychological-distress state			
(%)	No distress	Severe-depressive/borderline-anxiety distress	Comorbid severe anxiety/depressive/PTSD distress
No distress	**98.7**	1.2	0.1
Severe-depressive/borderline-anxiety distress	**56.5**	43.3	0.2
Comorbid severe anxiety/depressive/PTSD distress	30.2	**36.1**	33.8

Bold indicates the highest transition probability between different times; data on the diagonal indicate the probability of remaining in the same state between different times

### Associations between comorbid psychological-distress states and PGD at 6-month postloss

Among the 274 family surrogates who participated in 6-month-postloss survey, 268 provided PGD data, and 8 (3.0%) were categorized as with PGD. Surrogates’ distinct states of comorbid psychological distress estimated at 3-month postloss were significantly associated with their development of PGD at 6-month postloss (Table 4). The odds of development of PGD was higher for surrogates in the severe-depressive/borderline-anxiety-distress (OR [95% CI] = 14.58 [1.48, 143.54], *p* = 0.022) and the severe-anxiety/depressive/PTSD-distress states (104.50 [10.45, 1044.66], *p* < 0.001) in reference to the no-distress state.

**Table 4:** Prevalence of PGD at 6-month postloss across different psychological-distress states at 3-month postloss

Psychological-distress state at 3-month postloss	PGD at 6-month postloss					
	*n*	%	Odds ratio	95% CI		*p*
No distress (*n* = 210)	1	0.48	reference			
Severe-depressive/borderline-anxiety distress (*n* = 46)	3	6.52	14.58	1.48	143.54	.022
Severe anxiety/depressive/PTSD distress (*n* = 12)	4	33.33	104.50	10.45	1044.66	< .001

*PGD* prolonged grief disorder; *CI* confidence interval; *PTSD* post-traumatic stress disorder

## Discussion

We identified three distinct states of comorbid psychological distress among bereaved ICU surrogates with the majority (56.3–76.8%) retaining their psychological well-being as in the stable no-distress state. However, a minority of surrogates suffered severe-depressive and borderline-anxiety symptoms (30.5%) and severe comorbid symptoms of anxiety, depression, and PTSD (13.3%) when they first transited into bereavement and tended to transition into states of less psychological distress at 3-month postloss. Surrogates in the severe-depressive/borderline-anxiety-distress and severe-anxiety/depressive/PTSD-distress states at 3-month postloss were significantly more likely to develop PGD at 6-month postloss.

Our identification of the three distinct comorbid psychological-distress states within the first 3 months of bereavement confirms the common observations that family members heterogeneously experience grief reactions after the death of a loved one, but the majority are resilient [34, 42]. We also confirmed the conclusion made in the literature that severe grief reactions of bereaved family members of ICU decedents improve over time [6, 9, 12, 13]: family surrogates in the severe-depressive/borderline-anxiety-distress and the severe-anxiety/depressive/PTSD-distress states when they first transited into bereavement proceeded to states of less distress with only less than one-fourth of bereaved surrogates in these two comorbid psychological-distress states at the subsequent assessment.

Our novel identification of the severe-depressive/borderline-anxiety-distress and the severe-anxiety/depressive/PTSD-distress states contributes to the knowledge of comorbidity of psychological distress among bereaved family surrogates of ICU decedents. We observed unprecedently the co-occurrence of severe depressive symptoms exceeding the threshold with borderline anxiety symptoms approaching the threshold to support the well-established comorbidity of depression and anxiety [32] in the critical care literature. Most importantly, we extended the previous rare observations of co-occurrence of PTSD with depression [4, 16] or anxiety [4] for family members of ICU patients to the comorbidity of severe anxiety, depression, and PTSD symptoms, as recorded in the general population [32]. Healthcare professionals should recognize not only single, isolated emotional disturbances but should also be alert and responsive to the co-occurrence of multiple forms of psychological distress and their potential negative impacts on bereaved surrogates as evidenced by increased physical and psychological morbidity and all-cause mortality among those suffering comorbid psychological distress [43, 44].

Indeed, we exploratorily demonstrated that in reference to the no-distress state, the severe-depressive/borderline-anxiety-distress and the severe-anxiety/depressive/PTSD-distress states estimated at 3-month postloss increased surrogates’ subsequent development of PGD at 6-month postloss (Table 4). Building on previous evidence that severe depressive symptoms [9] or more PTSD symptoms [19] lead to PGD, our finding emphasizes the significant role played by comorbid severe symptoms of anxiety, depression, and PTSD in development of PGD. Considering the profound negative impacts of PGD on an individual’s physical-psycho-social well-being [25, 26], the unique support needs of bereaved surrogates who suffered comorbid severe symptoms of anxiety, depression, and PTSD should be identified as early as 1 month postloss and throughout the first 3 months of bereavement. Adequate bereavement services should be provided to appropriately aid these at-risk bereaved surrogates to prevent PGD.

The wide range of 95% CIs estimated for odds of experiencing PGD for the severe-depressive/borderline-anxiety-distress and severe-anxiety/depressive/PTSD-distress states at 6-month postloss may be attributable to the low prevalence of PGD. Overall PGD prevalence observed in this study (3.0%) is close to the 5% reported by Siegel and colleagues [5] but is substantially lower than the pooled prevalence of 9.8% (95% CI 6.8–14.0) from meta-analysis of 14 studies of adult populations exposed to non-violent bereavement [45] and those reported for bereaved family members of ICU decedents from Western countries (10.3% in Spain [21], 19% in Canada [19], and 21.7% [20], 23% [6], and 40% [18] in the USA, as well as 51.9% [12]–52.1% [9] in France).

Our observed low rate of PGD among Taiwanese bereaved family surrogates, as compared to those reported in studies from Western countries, may be due to cross-cultural differences in grief reactions. Grieving family members in more individualistic Western cultures may more independently adjust to losing a loved one with the comfort of religion [34] or support from their social network [46, 47]. Conversely, in Taiwanese culture, caring for a loved one is viewed as a family affair based on the concept of filial duty rooted in Confucian doctrines [48]. Family members in Asian cultures, who are strongly influenced by Confucian doctrines and are more family-oriented, tend to provide not only emotional but also practical and financial support [49] to help the bereaved family member adjust to the loss of a longstanding relationship and to start a new life without the decedent, thereby reducing the likelihood of suffering PGD. Besides, cultural norms for grief reactions may account for the low prevalence of PGD in our study. In Taiwanese culture, funeral ceremonies occur 7 days, 7 weeks, and 100 days after a loved one’s death, during which the bereaved are encouraged to publicly display their sorrow and grief, but after 100 days (as in the minimum duration criteria for PGD and the time for the 6-month-postloss survey), public displays of grief are not the social norm [50]. Because ritualized grieving is an evidence-based treatment for PGD [51], Taiwanese funerary tradition may substantially lower the prevalence of PGD. Indeed, prevalence of PGD at 6-month postloss was reported as 7.73% for Taiwanese bereaved family members of terminally ill cancer patients [52].

Several important limitations of our study were recognized. External validation of our findings in other national and international bereaved family samples is needed to support generalizability, especially considering cultural variations in grief reactions towards losing a loved one in Western and Asian countries [34, 48], Our results cannot be generalized to surrogates of patients who died within 3 days of ICU admission and with unnatural causes of death, or surrogates who did not participate in or withdrew from bereavement surveys. Family surrogates' psychological distress was evaluated by only one family surrogate per patient, despite a report of important variability among family members in rating the quality of death and dying [53] and potentially in experiences of psychological distress. Instruments used in this study are screening tools for psychological distress and not “gold standard” diagnostic measures, thereby likely overestimating bereaved surrogates’ psychological distress but avoiding overlooking their need for emotional support. Bereaved family surrogates may hide their prolonged, intensive grief reactions from researchers to conform to the social norm of restricting expression of their grief in public after 100 days from a loved one’s death, leading to underestimated prevalence of PGD symptoms. We cannot infer a causal relationship between the three distinct psychological-distress states and PGD, despite sequentially arranging the lagged comorbid psychological-distress states before PGD, and we recognize our analyses as exploratory in nature. Due to the low outcome-event rate, important covariates (e.g., surrogates’ age, gender, relationship with the patient, preexisting mental health and medical problems, prior loss experiences [including losses in ICUs], and involvement in EOL-care decision making during the patient’s ICU stays, as well as patient demographics, disease characteristics, and EOL-care received before the patients died in ICUs) were not controlled. Further validation of our findings in large studies with enough power and sufficient control of covariates to detect the different experiences of PGD across the three comorbid psychological-distress states is highly warranted. Factors predisposing bereaved surrogates to the distinct psychological-distress states have not yet been explored, but we will explore this issue in forthcoming studies.

## Conclusions and clinical implications

We identified three psychological-distress states to show comorbid severe symptoms of anxiety, depression, and PTSD among bereaved family surrogates of ICU decedents and demonstrated their evolution toward states of less distress over the first 3 months of bereavement. Our exploratory findings showed that being in the severe-depressive/borderline-anxiety-distress or severe-anxiety/depressive/PTSD-distress state at 3-month postloss was significantly associated with ICU family surrogates’ higher likelihood of developing PGD at 6-month postloss. Bereavement support is recommended as part of family-centered care in critical care settings [31]. Our findings highlight the urgent needs for intensivists to 1) understand that psychological distress not only occurs individually but also can co-occur as comorbid psychological-distress states, 2) identify bereaved family surrogates who may suffer comorbid severe symptoms of anxiety, depression, and PTSD at early bereavement (1–3-month postloss), and 3) develop effective bereavement interventions to appropriately address at-risk bereaved surrogates’ psychological distress before it evolves into PGD to facilitate successful bereavement adjustment for the betterment of individuals and society.

## Supplementary Information

**Additional file 1: Online Data Supplement 1:** Comparisons of family characteristics across participation status during bereavement follow-ups; **Online Data Supplement 2:** Model fit indexes for one- to four-state solutions of emotional distress; **Online Data Supplement 3:** Model fit figures for one- to four-state solutions of emotional distress; **Online Data Supplement 4:** Levels of symptoms of anxiety, depression, and PTSD across different comorbid-psychological-distress states.

## Data Availability

The sharing of anonymized data from this study is restricted due to ethical and legal constrictions. Data contain sensitive personal health information, which is protected under The Personal Data Protection Act in Taiwan, thus making all data requests subject to Institutional Review Board (IRB) approval. Per Chang Gung Memorial Hospital (CGMH) IRB, the data that support the findings of this study are restricted for transmission to those in the primary investigative team. Data sharing with investigators outside the team requires IRB approval. All requests for anonymized data will be reviewed by the research team and then submitted to the CGMH IRB for approval.

## Abbreviations

AIC: Akaike information criterion
APACHE: Acute physiology and chronic health evaluation
BIC: Bayesian information criterion
CI: Confidence interval
EOL: End of life
HADS: Hospital anxiety and depression scale
HADS-A: HADS anxiety subscale
HADS-D: HADS depression subscale
IC: Information criterion
ICU: Intensive care unit
IES-R: Impact of event scale-revised
LL: Log-likelihood
LTA: Latent transition analysis
OR: Odds ratio
PTSD: Post-traumatic stress disorder
PGD: Prolonged grief disorder
SABIC: Sample-size adjusted BIC

## Code availability

Specifications for Latent GOLD codes for statistical analyses are available from the corresponding and the first authors upon reasonable request.
