# Supplementary material for: ICU bereaved surrogates’ comorbid psychological-distress states and their associations with prolonged grief disorder
Source: Crit Care. 2022 Apr 11;26:102. doi: 10.1186/s13054-022-03981-7 (PMC8996508; doi:10.1186/s13054-022-03981-7)
Supplement: Supplementary file 1 — Additional file 1: Online Data Supplement 1: Comparisons of family characteristics across participation status during bereavement follow-ups; Online Data Supplement 2: Model fit indexes for one- to four-state solutions of emotional distress; Online Data Supplement 3: Model fit figures for one- to four-state solutions of emotional distress; Online Data Supplement 4: Levels of symptoms of anxiety, depression, and PTSD across different comorbid-psychological-distress states. [file 13054_2022_3981_MOESM1_ESM.docx]

**Online Data Supplement 1. Comparisons of family characteristics across participation status during bereavement follow-ups (*N* = 302)^a^**

| Variable | Participants  (*n*=260) | Skipped follow-up  (*n*=24) | Withdrew from follow-ups (*n*=18) | *P* |
| --- | --- | --- | --- | --- |
| Age, Mean(SD) |  |  |  | .225 |
|  | 49.78(12.72) | 48.13(10.44) | 54.56(11.76) |  |
| Gender, *n* (%) |  |  |  | .478 |
| Male | 105(40.4%) | 11(45.8%) | 5(27.8%) |  |
| Female | 155(59.6%) | 13(54.2%) | 13(72.2%) |  |
| Marital status, *n* (%) |  |  |  | .126 |
| Single | 56(21.5%) | 6(25.0%) | 1(5.6%) |  |
| Married/Cohabiting | 197(75.8%) | 18(75.0%) | 15(83.3%) |  |
| Separated/Widowed | 7(2.7%) | 0(0.0%) | 2(11.1%) |  |
| Educational level, *n* (%) |  |  |  | .820 |
| >High school | 128(49.2%) | 13(54.2%) | 8(44.4%) |  |
| ≦High school | 132(50.8%) | 11(45.8%) | 10(55.6%) |  |
| Financial status, *n* (%) |  |  |  | .697 |
| Making ends meet | 216(83.1%) | 21(87.5%) | 15(83.8%) |  |
| Financial strain | 39(15.0%) | 2(8.3%) | 2(11.1%) |  |
| Other | 5(1.9%) | 1(4.2%) | 1(5.6%) |  |
| Relationship, *n* (%) |  |  |  | .104 |
| Spouse | 79(30.4%) | 4(16.7%) | 7(38.9%) |  |
| Child | 135(51.9%) | 19(79.2%) | 8(44.4%) |  |
| Other | 46(17.7%) | 1(4.2%) | 3(16.7%) |  |
| Chronic disease, *n* (%) |  |  |  | .094 |
| Yes | 98(37.7%) | 4(16.7%) | 5(27.8%) |  |
| No | 162(62.3%) | 20(83.3%) | 13(72.2%) |  |
| Living with the patient, *n* (%) | |  |  | .934 |
| Yes | 172(66.2%) | 15(62.5%) | 12(66.7%) |  |
| No | 88(33.8%) | 9(37.5%) | 6(33.3%) |  |

| Variable | Participants  (*n*=260) | Skipped follow-up  (*n*=24) | Withdrawal from follow-ups (*n*=18) | *P* |
| --- | --- | --- | --- | --- |
| Hospitalization for mental health problems, *n* (%) | | | | .^b^ |
| Yes |  |  |  |  |
| No | 260(100.0%) | 24(100.0%) | 18(100.0%) |  |
| Hospitalization for medical problems, *n* (%) | | | | .334 |
| Yes | 13(5.0%) | 0(0.0%) | 0(0.0%) |  |
| No | 247(95.0%) | 24(100.0%) | 18(100.0%) |  |
| Emergency room visit, *n* (%) | |  |  | .692 |
| Yes | 19(7.3%) | 1(4.2%) | 2(11.1%) |  |
| No | 241(92.7%) | 23(95.8%) | 16(88.9%) |  |
| Medication use for pain problems, *n* (%) | | |  | .045 |
| Yes | 34(13.1%) | 0(0.0%) | 0(0.0%) |  |
| No | 226(86.9%) | 24(100.0%) | 18(100.0%) |  |
| Medication use for anxiety problems, *n* (%) | | |  | .515 |
| Yes | 8(3.1%) | 0(0.0%) | 0(0.0%) |  |
| No | 252(96.9%) | 24(100.0%) | 18(100.0%) |  |
| Medication use for depressive problems or other psychiatric disturbances, *n* (%) | | | | .250 |
| Yes | 2(0.8%) | 1(4.2%) | 0(0.0%) |  |
| No | 258(99.2%) | 23(95.8%) | 18(100%) |  |

SD: Standard deviation

^a^ Among the 319 family surrogates who participated in bereavement surveys, follow-up assessments were not due for 17 participants, and one participant skipped twice (Figure 1).

^b^ Cannot be estimated.

**Online Data Supplement 2**. Model fit indexes for one- to four-state solutions of emotional distress

| State number | Log likelihood | BIC | AIC | SABIC | Number of parameters | Degrees of freedom | Classification Error | Entropy |
| --- | --- | --- | --- | --- | --- | --- | --- | --- |
| 1 | -820.706 | 1658.689 | 1647.413 | 1649.174 | 3 | 136 | 0.0000 |  |
| 2 | -603.710 | **1259.251** | 1225.421 | 1230.705 | 9 | 130 | 0.0566 | 0.783 |
| 3 | -580.746 | 1259.393 | **1195.492** | **1205.473** | 17 | 122 | **0.0545** | **0.793** |
| 4 | -577.331 | 1310.153 | 1208.663 | 1224.516 | 27 | 112 | 0.1043 | 0.701 |

AIC= Akaike information criterion; BIC= Bayesian information criterion; SABIC=sample-adjusted BIC.

Bolds indicate the best fit indices

**Online Data Supplement 3**. Model fit figures for one- to four-state solutions of emotional distress

LL Values

ICs

IC Values

ICs

IC: information criterion; LL: log-likelihood; AIC: Akaike information criterion; BIC: Bayesian information criterion;

SABIC: sample-size adjusted BIC.

**Online Data Supplement 4**. Levels of symptoms of anxiety, depression, and PTSD across different comorbid-psychological-distress states

| Emotional distress  Mean (SD) | Comorbid psychological-distress state | No distress | Severe-depressive/borderline-anxiety distress | Comorbid severe anxiety/depressive/PTSD distress |
| --- | --- | --- | --- | --- |
| 1 month postloss (n) | | 173 | 100 | 36 |
| Anxiety symptoms^a^ | | 2.79 (1.61) | 7.09 (3.10) | 11.17 (4.10) |
| Depressive symptoms^b^ | | 3.96 (2.08) | 10.04 (2.42) | 13.50 (3.64) |
| PTSD symptoms^c^ | | 14.92 (8.42) | 14.92 (8.42) | 43.20 (9.33) |
| 3 months postloss (n) | | 233 | 53 | 12 |
| Anxiety symptoms^a^ | | 2.45 (1.72) | 7.43 (3.49) | 12.50 (5.11) |
| Depressive symptoms^b^ | | 3.59 (2.44) | 10.23 (2.34) | 14.75 (3.70) |
| PTSD symptoms^c^ | | 5.06 (4.39) | 15.23 (8.42) | 41.73 (5.53) |

SD: Standard deviation

PTSD: post-traumatic stress disorder

a: measured by the Hospital Anxiety and Depression Scale (HADS)-Anxiety subscale; b: measured by the HADS-Depression subscale; c: measured by the Impact of Event Scale-Revised.
